# Evaluation of novel rapid detection kits for dengue virus NS1 antigen in Dhaka, Bangladesh, in 2017

**DOI:** 10.1186/s12985-019-1204-y

**Published:** 2019-08-15

**Authors:** Keita Suzuki, Emi E. Nakayama, Akatsuki Saito, Akio Egawa, Tairyu Sato, Juthamas Phadungsombat, Rummana Rahim, Abu Hasan, Hisahiko Iwamoto, Mizanur Rahman, Tatsuo Shioda

**Affiliations:** 1 Department of Viral Infections, Research Institute for Microbial Diseases, Osaka University, 3-1, Yamada-oka, Suita, Osaka, 565-0871 Japan; 2 POCT Business Unit, TANAKA Kikinzoku Kogyo K.K, 2-73, Shinmachi, Hiratsuka, Kanagawa 254-0076 Japan; 3 Mahidol-Osaka Center for Infectious Diseases, Mahidol University, 420/6 Ratchawithi road, Ratchathewi, Bangkok, 10400 Thailand; 4 Apollo Hospitals Dhaka, Plot-81, Block-E, Bashundhara R/A, Dhaka, 1229 Bangladesh

**Keywords:** Dengue, DENV, NS1, IC, POCT, RDT

## Abstract

**Background:**

Dengue virus (DENV) infection is one of the biggest challenges for human health in the world. In addition, a secondary DENV infection sometimes causes dengue hemorrhagic fever (DHF), which frequently leads to death. For this reason, accurate diagnosis record management is useful for prediction of DHF. Therefore, the demand for DENV rapid diagnosis tests (RDTs) is increasing because these tests are easy and rapid to use. However, commercially available RDTs often show low sensitivity for DENV and cross-reactivity against other flaviviruses, especially Zika virus (ZIKV).

**Methods:**

We developed two types of novel DENV non-structural protein 1 (NS1) detection RDTs, designated TKK-1st and TKK-2nd kits. Specificities of the monoclonal antibodies (MAbs) used in these kits were confirmed by enzyme-linked immuno-sorbent assay (ELISA), dot blot, and western blot using recombinant NS1 proteins and synthetic peptides. For evaluation of sensitivity, specificity, and cross-reactivity of the novel DENV NS1 RDTs, we first used cultured DENV and other flaviviruses, ZIKV and Japanese encephalitis virus (JEV). We then used clinical specimens obtained in Bangladesh in 2017 for further evaluation of kit sensitivity and specificity in comparison with commercially available RDTs. In addition, RNA extracted from sera were used for viral genome sequencing and genotyping.

**Results:**

Epitopes of three out of four MAbs used in the two novel RDTs were located in amino acid positions 100 to 122 in the NS1 protein, a region that shows low levels of homology with other flaviviruses. Our new kits showed high levels of sensitivity against various serotypes and genotypes of DENV and exhibited high levels of specificity without cross-reactivity against ZIKV and JEV. In clinical specimens, our RDTs showed sensitivities of 96.0% (145/151, TKK-1st kit) and 96.7% (146/151, TKK-2nd kit), and specificities of 98.0% (98/100, TKK-1st kit and TKK-2nd kit). On the other hand, in the case of the commercially available SD Bioline RDT, sensitivity was 83.4% (126/151) and specificity was 99.0% (99/100) against the same clinical specimens.

**Conclusions:**

Our novel DENV NS1-targeting RDTs demonstrated high levels of sensitivity and lacked cross-reactivity against ZIKV and JEV compared with commercially available RDTs.

**Electronic supplementary material:**

The online version of this article (10.1186/s12985-019-1204-y) contains supplementary material, which is available to authorized users.

## Background

Dengue virus (DENV) infection is one of the most common tropical infectious diseases transmitted through mosquitoes to human [1]. DENV infection is currently endemic in more than 110 countries, and the number of DENV-infected patients has been estimated to be 390 million per year [2], resulting in 20 thousand patient deaths per year [3, 4]. The symptoms of DENV infection are high fever, headache, muscle and joint pains, and rash. There are four serotypes of DENV, which are referred to as DENV-1, DENV-2, DENV-3, and DENV-4. All four serotypes are known to cause dengue fever (DF) [5].

Although DENV infection is a big challenge for human health, no antiviral drugs targeting DENV have been approved to date. On the other hand, the first preventive vaccine for DENV infection has been approved [6], but, due to frequent occurrence of severe DENV cases in previously uninfected vaccines, this vaccine can be used only in previously DENV-infected cases [7]. Generally, primary DENV infection causes DF, whereas a small but non-negligible proportion of patients with secondary infection manifest the more severe symptoms of dengue hemorrhagic fever (DHF), which can cause death [8]. It has been suggested that antibodies against one serotype of primary DENV enhance the replication of other serotypes of DENV upon secondary infection, causing the more severe symptoms seen with DHF [9]. Therefore, it is important to obtain accurate patient records regarding previous DENV infection to predict the severity of ensuing disease, permitting clinical risk management of DHF. Easy access to rapid diagnosis would facilitate the generation of accurate records of DENV infection.

Currently, there are a variety of methods for diagnosis of DENV infection such as RNA-based assays (real-time RT-PCR) and tests that detect the DENV non-structural protein (NS1) antigen and/or patient IgM/IgG (rapid detection test and ELISA) [10]. Among these diagnostic assays, the rapid detection test (RDT) is the easiest method for diagnosis of virus infection. RDTs are user-friendly and cost-effective, require no equipment, and provide results faster than PCR and ELISA. The flavivirus NS1 protein is a glycoprotein of approximately 46–50 kDa, highly conserved among the 4 DENV serotypes and even among arthropod-borne flaviviruses (reviewed in [11]). NS1 protein is a useful infection marker for rapid diagnosis because this protein is released from infected cells into the blood stream [12], accumulating in the blood of DENV-infected patients at concentrations of up to 50 μg/mL [13]. Accordingly, NS1 protein persists longer than the viral genome in blood [12, 14–19]. Even after DENV RNA in patient blood has been degraded, NS1 protein can still be detected by RDTs. Although NS1-detecting RDTs provide useful and critical diagnostic information, these tests can cause misdiagnosis as a result of cross-reaction [14, 20, 21] or nonspecific reaction [14, 16, 22]. Most cross-reactions are caused by the similarity among flavivirus NS1 proteins. The flavivirus NS1 proteins are highly conserved among the genus flavivirus [11, 12], and commercially available RDTs have been shown to exhibit cross-reactivity with the NS1 proteins of other flaviviruses [21], pathogens that are endemic to geographic regions that overlap with the distribution of DENVs [1, 2].

RDTs have several disadvantages such as lower sensitivity and specificity than RT-PCR-based assays [20, 23–26]. Notably, RDTs for DENV antigen and antibody demonstrate cross-reactivity against Zika virus (ZIKV), another flavivirus that causes symptoms similar to those caused by DENVs [21, 27, 28]. Furthermore, patient sera might contain antibodies against DENV if patients have come to hospitals later than 7 days after the onset of fever or if patients have been infected previously with DENV. Such pre-existing antibodies would compete with the monoclonal antibodies (MAbs) of RDTs [29] and interfere with the reaction monitored by RDTs. RDT signals upon secondary infection usually are weaker than those detected upon primary infection [14, 15, 20, 23, 25, 26, 30, 31]. Therefore, it would be desirable to have more-sensitive and less-cross-reactive antigen-detecting RDTs for accurate DENV diagnosis.

Here, we report novel DENV NS1 antigen detection kits that showed high sensitivity to DENV patient sera in Bangladesh, a dengue-endemic country [32]. The kits showed no cross-reactivity against ZIKV and Japanese encephalitis virus (JEV).

## Materials and methods

### Assembly of rapid detection test (RDT)

Twelve mouse MAbs against DENV NS1 protein were purchased from Bio Matrix Research, Inc. Nagareyama, Japan (Catalog No. BMRdn). Each anti-DENV NS1 MAb was conjugated with gold nanoparticles (AuNPs) (TANAKA Kikinzoku Kogyo K. K, Hiratsuka, Japan) according to the previously described method [33]. After washing three times with phosphate-buffered saline (PBS) containing 1% bovine serum albumin (BSA), the AuNP-conjugated MAb was diluted to an optical density of 2.5 and impregnated into glass fibers (Millipore, Billerica, MA), which then were dried in an incubator. To prepare the detection matrix, anti-DENV NS1 antibodies were diluted to 0.5 mg/mL and immobilized onto a nitrocellulose membrane to permit capture of the DENV NS1 protein at a test line. Anti-Mouse IgG antibody (Protein Purify Industrial Co.,LTD, Isesaki,Japan) also was immobilized onto the nitrocellulose membrane along a control line. RDTs (dipstick format) were generated by assembling a glass fiber (sample pad), conjugate pad, nitrocellulose membrane, liquid absorbent pad, and laminate tape. Extraction buffer (including surfactant) was distributed to a 96-well plate at 60 μL/well; an aliquot (30 μL) of specimen was added to a given well and the contents were mixed gently. The RDT strip then was inserted into the well containing the mixture of extraction buffer and specimen. After 15 min, the color intensity of the test line was measured using an Immunochromato-Reader (Hamamatsu Photonics, Model C10088–10).

### Peptide-competitive ELISA of NS1 antigen

For epitope analysis of MAbs, we prepared recombinant NS1 from each of the four serotypes of DENV (Fitzgerald Industry International, North Acton, MA) and 7 synthetic peptides (Hokkaido System Science Co.,Ltd., Sapporo, Japan) corresponding to fragments of DENV-1 NS1, including amino acids 13 to 36 (P1), 100 to 122 (P2), 135 to 151 (P3), 193 to 222 (P4), 248 to 272 (P5), 282 to 306 (P6), and 325 to 352 (P7). These peptides were designed based on the predicted hydrophilicity of portions of the DENV-1 NS1 protein.

Amino acid sequence information for each recombinant NS1 protein was obtained from the NCBI database (DENV-1: clone 45AZ5, Accession No. NC_001477; DENV-2: S1 vaccine strain, Accession No. NC_001474; DENV-3: H87 strain, Accession No. NC_001475; DENV-4: clone rDEN4, Accession No. NC_002640).

Recombinant DENV-1 NS1 protein was diluted with 50 mM carbonate buffer (pH 9.5) to a concentration of 20 μg/mL, added to an ELISA plate (Nunc immune module F8 Maxi sorp, Thermofisher. Inc., USA), and incubated overnight at 4 °C. Wells were blocked with 0.1% BSA in 50 mM carbonate buffer for 1 h at 37 °C. After 3 washes with PBS containing 0.05% Tween-20 (PBS-T), 100 μL of MAb against DENV NS1 protein with each synthesized peptide (500 μg/well) were added to each well, and the plate was incubated for 1 h at 37 °C. After 3 washes with PBS-T, 100 μL of secondary antibody (anti-mouse IgG-peroxidase (POD) conjugate, Jackson Immuno Research Laboratory, Inc., USA.; formulated at 1: 1000 in PBS) was added to each well and the plate was incubated for 1 h at 37 °C. After three washes with PBS-T, 75 μL of 3,3′,5,5′-tetramethylbenzidine (TMB) solution was added to each well and the plate was incubated for 5 min at room temperature (RT). The absorbance at 650 nm of the solution in each well was measured using a microplate reader (EnSpire™, Perkin Elmer, Co., Ltd., USA). Wells with MAb only served as positive controls; plates with each synthesized peptide only served as negative controls.

### Dot blotting

DENV-2 NS1 recombinant protein was diluted with 50 mM carbonate buffer (pH 9.5) to concentrations of 20, 10, and 5 μg/mL. Aliquots (30 μL) of diluted NS1 were spotted to nitrocellulose membranes (#1620090, Bio-Rad Laboratories, Inc., Hercules, CA) to generate dots. To generate a dot lacking antigen, 0.1% BSA in 50 mM carbonate buffer was used. Nitrocellulose membranes were incubated for 1 h at RT and blocked with 0.1% BSA in 50 mM carbonate buffer for 1 h at RT. After 3 washes with PBS-T, 30 μL of each MAb (formulated at 20 μg/mL in PBS-T) was added and the membranes were incubated for 1 h at RT. After 3 washes with PBS-T, nitrocellulose membranes were dipped into 4 mL of POD-conjugated anti-mouse IgG for 1 h at RT with gentle shaking. After another 3 washes with PBS-T, the membranes were dipped into TMB for visualization and reactions were quenched with water after 10 s.

### Western blotting

DENV-1 and DENV-2 NS1 recombinant proteins were reduced by reaction with tris (2-carboxyethyl) phosphine (TCEP) and then loaded at 125 ng/lane and separated by SDS-PAGE using a 5–20% gradient polyacrylamide gel (XV PANTERA GEL MP, D.R.C. Co., Ltd., Tama, Japan). Separated NS1 proteins then were transferred to a nitrocellulose membrane using a semidry transfer method (180 mA for 45 min in transfer buffer (24 mM Tris, 77 mM glycine, 20% methanol)). Membranes were blocked with 4% skim milk in 50% PBS-T for 1 h at RT and sliced to yield strips containing of two lanes each (molecular marker and sample-loaded lane). After blocking, NS1 recombinant protein on the strips was visualized using a procedure similar to that described above for dot blotting.

### Clinical specimens

As a routine assay for febrile patients visiting the Apollo Hospitals Dhaka, whole blood samples (3 mL from adults, 0.5–1 mL from pediatric patients) were collected from patient exhibiting clinical symptoms indicative of either infection with chikungunya or dengue. Viral RNA was extracted from 200 μL serum using the QIAamp MinElute Virus Spin Kit (Qiagen, Hilden, Germany) according to manufacturer’s protocol. RNA was either assayed immediately or stored at − 80 °C pending analysis. The CE-IVD-approved commercial one-step reverse transcriptase real-time PCR kit (Fast Track Diagnostics, Luxembourg) was used for detection of DENV; the Genesig one-step reverse transcriptase real-time PCR kit (Primerdesign, Southanpton, UK) was used to determine the DENV serotypes. These PCR kits were used according to the respective kit manufacturers’ instructions; reactions were run on a Qiagen Rotor Gene Q thermocycler (Rahman et al. 2018). De-identified stored serum and RNA samples (each with distinct codes) that had been stored at − 80 °C were used for this research study.

### Detection of NS1 antigen

The recombinant NS1 proteins of the four serotypes of DENV, as well as those from JEV, and Zika virus, were purchased from Fitzgerald Industry International (DENV) or Meridian Life Sciences (JEV and ZIKV). The recombinant NS1 proteins of the four DENV serotypes were diluted in PBS to concentrations of 63, 32, 16, 8, 4, 2, 1, or 0.5 ng/mL. DENV strains DENV-1 (Mochizuki strain), DENV-2 (16681 strain), DENV-3 (H87 strain), and DENV-4 (H241 strain), as well as clinical isolates B17-1387DV1, B17-1547DV1, B17-1431DV2, B17-1482DV2, B17-1552DV2, B17-1634DV2, B17-1471DV3, and B17-1479DV3 (Suzuki et al., manuscript in preparation) were propagated in Vero cells; virus titers in culture supernatants were determined by real-time PCR [34]. JEV (Nakayama strain) and Zika virus (MR766 strain) were propagated in Vero and C6/36 cells, respectively, and virus titers were determined by the plaque formation assay.

Aliquots (30 μL) of serially diluted recombinant NS1 proteins, serially diluted virus stock, or undiluted patient sera were mixed with 60 μL of dilution buffer. The chromatographic dipsticks then were inserted into wells containing diluted samples and incubated for 15 min at RT. The test band intensities were measured by a chromatogram reader (Hamamatsu Photonics, Model C10088–10). Band intensities exceeding 15 m-absorbance units were visible to the naked eye. The SD-Bioline Duo kit (NS1Ag + IgG/IgM, Alere Medical, Waltham, MA) and PanBio Dengue Early Rapid antigen kit (Abbott Inc., Chicago, IN) were used according to respective manufacturer’s instructions. NS1 antigen ELISA (DENV Detect InBios, Seattle, WA) was performed according to the manufacturer’s instruction. Immune Status Ratio (ISR) was calculated from the optical density of the test sample divided by the calculated cut-off value.

### RNA extraction and genome amplification

The NS1-encoding regions of DENV genomic RNA were amplified using the One-step RT-PCR kit (Qiagen, Hilden, Germany) with primers for each serotype. Amplification was performed under the following conditions: reverse transcription at 50 °C for 30 min; inactivation of the reverse transcriptase enzyme at 95 °C for 15 min; 35 cycles of denaturation at 94 °C for 30 s, annealing at 55 °C, 46 °C, or 49 °C for DENV-1, − 2, or − 3, respectively, for 1 min, and extension at 72 °C for 2 min and 30 s; and a final extension step at 72 °C for 10 min. Amplified products then were subjected to nested PCR with primers (Additional file 1) targeting a 2.0-kb region of the NS1-encoding gene [35]. Gel electrophoresis was performed using a 0.8% agarose gel at 100 V for 30 min. After electrophoresis, the size of the target DNA was confirmed by comparison to a DNA ladder, and the portion of the gel containing the target DNA was excised using a razor. The QIAquick Gel Extraction Kit (Qiagen, Hilden, Germany) was used according to the manufacturer’s protocol to purify DNA from the agarose. The recovered DNA was subjected to sequencing using BigDye terminator 3.1 Cycle Sequencing with the *NS1* sequencing primers (listed in Additional file 1) under the following conditions: denaturation at 96 °C for 1 min; 25 cycles of denaturation at 96 °C for 10 s, annealing at 50 °C for 5 s, and extension at 60 °C for 4 min; and then run on an ABI Model 3130XL DNA sequencer. Phylogenetic trees were inferred from the alignment using the maximum-likelihood approach generated in W-IQ-TREE [36], http://iqtree.cibiv.univie.ac.at/. The best-fit model was selected by ModelFinder [37], and an ultrafast bootstrap [38] with 1000 replicates was calculated.

### Ethical clearance

The study proposal was approved by the Research and Ethical Practice Committee of Apollo Hospitals Dhaka (Approval No. ERC 16/2018–3).

## Results

### Selection of MAbs against DENV-NS1 for RDT

To develop an NS1 antigen detection RDT, we obtained 12 MAbs against DENV-NS1 from Bio Matrix Research, Inc. To increase testing throughput, we used a dipstick format, which permitted completion of each test in approximately 15 min (depending on humidity conditions). We conjugated the MAbs with AuNPs, adsorbed the MAbs onto nitrocellulose membranes, and tested all combination of MAbs by placing dipsticks in microcentrifuge tubes or the wells of a 96-well plate containing 60 μL of extraction buffer and 30 μL of sample specimen per tube or well. First, we tested recombinant NS1 proteins of all four serotypes of DENV and selected 12 combinations that showed signal/background ratios greater than 2.0. The high percentage of amino acid homology and identity among flavivirus NS1 proteins has been reported previously [39]. Therefore, we tried to select MAb combinations that did not show cross-reactivity with 100 ng/mL recombinant NS1 from JEV or Zika (Meridian Life Science, Inc. Memphis, TN). Based on the results of these screenings of MAbs, 4 MAbs (designated Membrane-1, Membrane-2, AuNP-1, and AuNP-2) were selected.

**Fig. 1:**
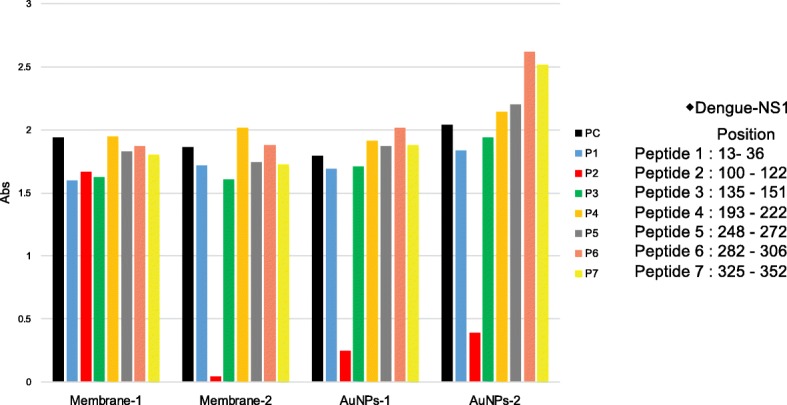
Competitive ELISA using synthetic peptides. The recombinant DENV-1 NS1 proteins (20 μg/mL) were coated on an ELISA plate. Aliquots (2 μg/well) of each antibody (Membrane-1, Membrane-2, AuNPs-1 and AuNPs-2) were mixed with each peptide (500 μg/well) corresponding to the amino acid positions shown in the left panel. Peroxidase-conjugated anti-mouse IgG was used to detect antibodies bound to NS1 protein. Abs. indicates the optical density at 650 nm. P1: peptide-1, P2: peptide-2, P3: peptide-3, P4: peptide-4, P5: peptide-5, P6: peptide-6, and P7: peptide-7. PC: positive control (run without added synthetic peptide)

Next, we performed a competitive inhibition enzyme-linked immunosorbent assay (ELISA) using recombinant full-length DENV-NS1 and synthetic DENV-NS1 peptides designated P1 to P7. As shown in Fig. 1, the reactions of NS1 protein with MAbs were inhibited by the addition of P2; the sole exception was the reaction with the Membrane-1 MAb. Therefore, three of the MAbs (Membrane-2, AuNP-1, and AuNP-2) recognized DENV-NS1 via epitopes corresponding to amino acids 100 to 122. Since the reaction of DENV-NS1 with Membrane-1 was not inhibited by adding any synthesized peptide, it was speculated that the Membrane-1 MAb recognizes a conformation of NS1 or a hydrophilic region of NS1 protein other than the regions covered by the 7 tested peptides. Indeed, the Membrane-1 MAb did not detect DENV-NS1 by western blotting under reducing conditions but did detect DENV-NS1 by dot blotting under non-reducing condition. On the other hand, the other 3 MAbs detected DENV-NS1 by both western blotting (reducing conditions) and dot blotting (non-reducing conditions). (Additional file 2). Based on these results, we assembled 2 types of RDTs in dipstick format, which we designated as TKK-1st and TKK-2nd kits. The TKK-1st kit utilized paired MAbs Au NPs-1 and Membrane-1, while the TKK-2nd kit utilized paired MAbs Au NPs-2 and Membrane-2.

### Detection of recombinant NS1 proteins and culture supernatant of DENV laboratory strains by RDTs

**Fig. 2:**
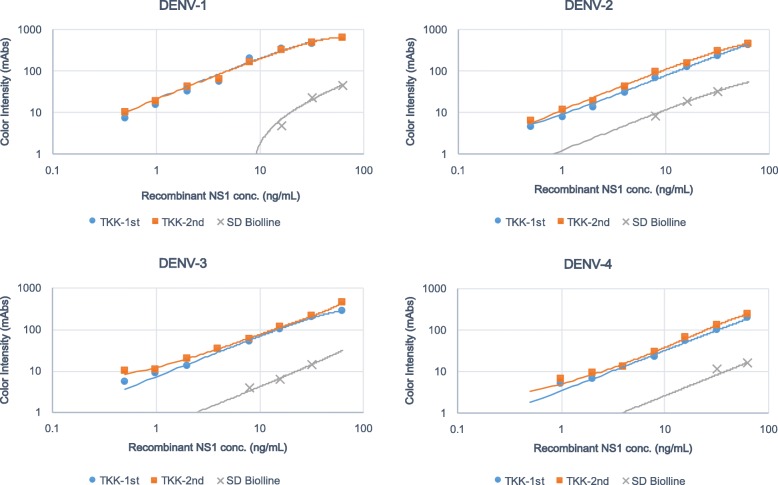
Detection of recombinant NS1 proteins of DENV-1 to − 4 by RDTs. Recombinant DENV NS1 proteins were serially diluted in PBS. The lower limits of detection for the RDTs were defined by chromatographing dilutions of the recombinant NS1 proteins and then quantifying the signal color intensities (mAbs: milli-absorbance units). Blue circles, red squares, and gray crosses indicate results of the TKK-1st kit, the TKK-2nd kit, and a SD Bioline kit, respectively

Recombinant NS1 proteins of all 4 serotypes of DENV were prepared at 63 ng/mL and serially diluted in PBS. We determined the limit of visible detection (i.e., with the naked eye) of NS1 protein as 15 m-absorbance units (mAbs) of color intensity as measured by an Immunochromato-Reader. The lower limit of detection of the TKK-1st and -2nd kits was 1 ng/mL of DENV-1, 2 ng/mL of DENV-2 and DENV-3, and 8 ng/mL of DENV-4 NS1 proteins. On the other hand, the lower limit of detection of a commercially available SD Bioline kit was 32 ng/mL of DENV-1 and DENV-3, 16 ng/mL of DENV-2, and 63 ng/mL of DENV-4 NS1 proteins (Fig. 2).

**Fig. 3:**
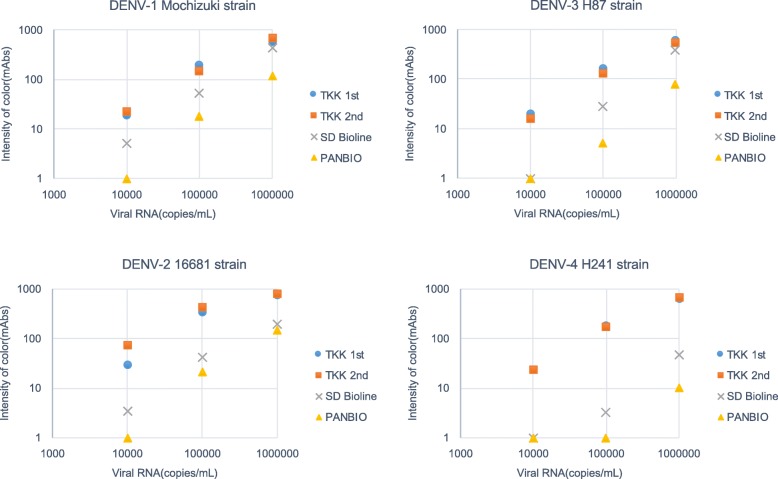
Detection of NS1 in culture supernatants of laboratory strains (DENV-1 to 4) by RDTs. Culture supernatants from Vero cells infected with each serotype of DENV (DENV-1: Mochizuki strain, DENV-2: 16681 strain, DENV-3: H87 strain, DENV-4: H241 strain) were chromatographed on each RDT. The vertical axis indicates intensity of color (mAbs) measured by an Immunochromato-Reader; the horizontal axis indicates virus concentration (RNA copies/mL) based on RT-PCR. Blue circles, red boxes, gray crosses, and yellow triangles indicate results of the TKK-1st kit, TKK-2nd kit, SD Bioline kit, and PanBio kit, respectively

Next, we prepared DENV laboratory strains, including the Mochizuki strain of DENV-1, the 16681 strain of DENV-2, the H87 strain of DENV-3, and the H241 strain of DENV-4. Virus titers were determined by RT-PCR of viral RNA, and virus samples were serially diluted. Results showed that the TKK-1st kit and -2nd kit detected 1 × 10^4^ copies/mL of DENV-1 to DENV-4, while the SD Bioline kit failed to detect viruses at this dilution (Fig. 3). The PanBio Dengue Rapid kit failed to detect 1 × 10^5^ copies/mL of DENV-3 and DENV-4. These results indicated that the detection limits of the TKK-1st and -2nd kits were lower than those of the SD Bioline and PanBio kits.

**Fig. 4:**
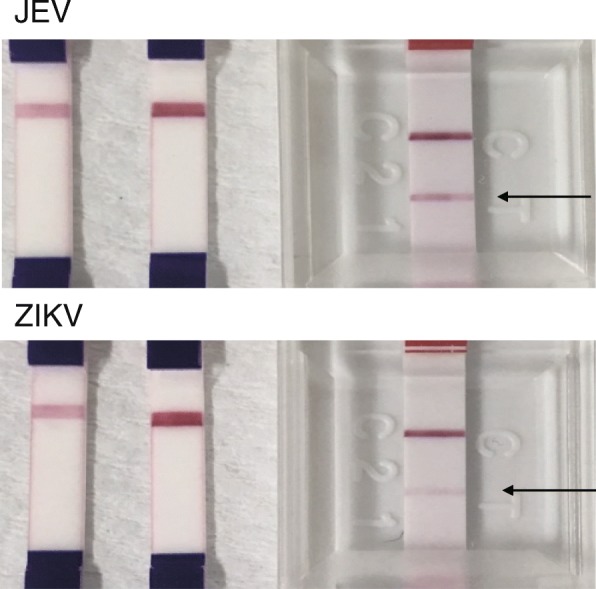
Cross-reactivity against other flaviviruses. Photos of results obtained with the TKK-1st kit (left), TKK-2nd kit (center), and SD Bioline kit (right) with JEV (Nakayama strain at 1 × 10^9^ focus-forming units/mL) and ZIKV (MR766 strain at 4.0 × 10^7^ plaque-forming units/mL) are shown. Arrows indicate positive signals at the position of the test line of the SD Bioline kit

Previous studies showed that DENV NS1 antigen rapid tests demonstrate limited cross-reactivity with other flaviviruses [20, 21]. We prepared Zika virus (MR766 strain) at 4.0 × 10^7^ plaque-forming units/mL and JEV (Nakayama strain) at 1 × 10^9^ focus-forming units/mL. Even with the high viral titers in those culture supernatants, the TKK-1st kit and -2nd kit did not exhibit any signals with either ZIKV or JEV, in contrast to the cross-reaction seen with the SD Bioline kit (Fig. 4).

### Detection of clinical isolates of DENVs by RDTs

**Fig. 5:**
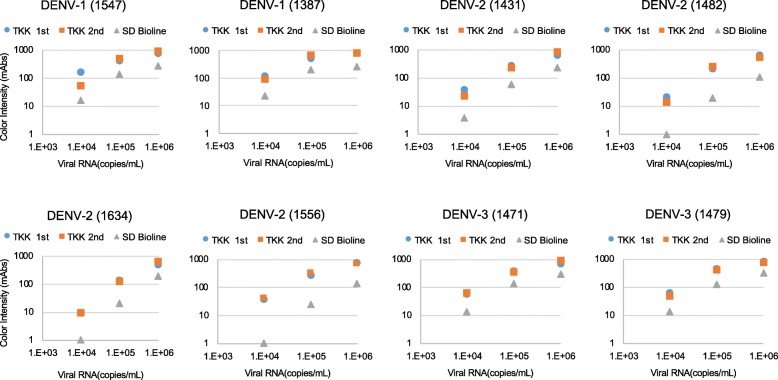
Detection of clinical DENV isolates from Bangladesh. Culture supernatants of Vero cells infected with clinical isolates (DENV-1: 1387 and 1547; DENV-2: 1431, 1482, 1556, and 1634; DENV-3: 1471 and 1479) isolated from serum collected in 2017, at Apollo Hospitals Dhaka, Bangladesh, were serially diluted, and applied to RDTs. The y-axis indicates intensity of color (mAbs) measured by an Immunochromato-Reader and the x-axis indicates virus concentration (RNA copies/mL) based on RT-PCR. Blue, red, and gray circles indicate results of the TKK-1st kit, TKK-2nd kit, and SD Bioline kit, respectively

To confirm the sensitivity of the RDTs to recently circulating DENVs, the culture supernatants of clinical isolates from DENV patients in Bangladesh in 2017 were used for evaluation of RDTs. We evaluated 2 isolates of DENV-1, 4 of DENV-2, and 2 of DENV-3. As seen with laboratory strains, the TKK-1st kit and -2nd kit detected signals in the culture supernatants containing 1 × 10^4^ copies/mL of each of the 6 clinical strains; the SD Bioline kit detected signal in only 2 of the 6 clinical strains at this dilution (Fig. 5). These results suggested that both the TKK-1st kit and TKK-2nd kit are capable of detecting recent clinical DENV isolates at sensitivities similar to those seen with laboratory strains.

### Patient samples

A total of 269 patients were diagnosed as infected by DENV (as assessed by real-time RT-PCR with a commercial primer and probe set (Fast-track Diagnostics, Esch-sur-Alzette, Luxembourg) between July 2017 to February 2018 at Apollo Hospitals Dhaka, Bangladesh. The median Ct value of all 269 DENV-infected samples was 24.03. For further analysis using the TKK kits, we used a total of 251 stored sera available as volumes of greater than 0.2 mL, including 151 samples that were DENV-PCR-positive and 100 that were DENV-PCR-negative. Among the 151 DENV-PCR-positive patients, the median age was 30 years (range, 1–79 years); 88 were males and 63 were females; 70 of the samples were collected from hospitalized patients and 81 were collected from a fever clinic. Serological information for these samples was obtained using a rapid antibody detection kit (SD Bioline Dengue IgG/IgM). Among the 251 total sera (including both DENV-positive and -negative samples), 63 were IgM- and/or IgG-positive sera (31 PCR-positive and 32 PCR-negative), and 188 were both IgM- and IgG-negative (120 PCR-positive and 68 PCR-negative). Unfortunately, information regarding the day of sample collection relative to fever onset was not available.

### Evaluation of RDTs using clinical samples

In a first experiment, we used 74 samples collected between October 2017 and February 2018 (Experiment 1). Fifty-seven DENV-PCR-positive sera were tested; 17 CHIKV-PCR-positive sera served as DENV-negative controls. Median Ct value of DENV-PCR in the 57 DENV-positive samples was 23.9, similar to the median Ct value (24.03) obtained across all 269 DENV-positive samples. The TKK-2nd kit demonstrated the highest sensitivity of 93.0%, followed by the TKK-1st kit (91.2%) and the SD Bioline kit (80.7%). The specificities were 100% for all kits (Table 1), indicating that the DENV NS1 antigen detection kit did not cross-react with CHIKV.

**Table 1:** Evaluation of RDTs in Experiment 1

		IC kit results				
Exp.1	PCR results	+	–	Sensitivity(%)	Specificity(%)	OAA(%)^a^
TKK-1st	+	52	5	91.2	100	93.2
(*n* = 74)	–	0	17			
TKK-2nd	+	53	4	93	100	94.6
(*n* = 74)	–	0	17			
SD Ag	+	46	11	80.7	100	85.1
(*n* = 74)	–	0	17			

^a^*OAA* Overall agreement

*SD Ag* SD Bioline dengue NS1 Ag

The sensitivities of these kits increased when we omitted anti-DENV antibody-positive specimens. Specifically, the sensitivities of the TKK-2nd, TKK-1st, and SD Bioline kits in 53 IgM-, IgG-negative samples were 95.2, 97.6 and 85.7%, respectively (Table 2). Among the remaining sera, three were positive for IgM only, 10 were positive for IgG only, and 8 were positive for both IgM and IgG.

**Table 2:** Evaluation of RDTs with IgM-, IgG-negative samples in Experiment 1

		IC kit results				
Exp.1	PCR results	+	–	Sensitivity(%)	Specificity(%)	OAA(%)^a^
TKK-1st	+	40	2	95.2	100	96.2
(*n* = 53)	–	0	11			
TKK-2nd	+	41	1	97.6	100	98.1
(*n* = 53)	–	0	11			
SD Ag	+	36	6	85.7	100	88.7
(*n* = 53)	–	0	11			

^a^*OAA* Overall agreement

*SD Ag* SD Bioline dengue NS1 Ag

Among the 57 cases tested in Experiment 1, 55 had RNA samples available for serotyping PCR. Of those 55, two were DENV-1, 46 were DENV-2, and 2 were DENV-3; serotype could not be determined for the remaining 5 samples due to low viral titers.

In a second experiment, we used samples collected between July 2017 to October 2017 (Experiment 2). Prior to this experiment, we tested the DENV-positive samples for serotype. These 94 DENV-positive samples consisted of 84 DENV-2, 4 DENV-1, and 2 DENV-3; serotypes were not determined for the remaining 4 samples. When we serotyped these specimens, we did not test specimens with high Ct values (i.e., low viral load) as measured by DENV/CHIKV RT-PCR. Therefore, the median Ct value of the 94 DENV-PCR-positive samples tested in Experiment 2 was 20.6, a value lower than the median Ct value (24.03) obtained across all 269 DENV-positive samples (Additional file 3). Negative control samples for Experiment 2 were randomly selected from sera that tested negative for both DENV and CHIKV by RT-PCR. The TKK-1st and -2nd kits demonstrated high levels of sensitivity (98.9%), yielding values that exceed that of the SD Bioline kit (86.2%). The specificities of RDTs for the TKK-1st and -2nd kits were 97.6%, and that of the SD Bioline kit was 98.8% (Table 3).

**Table 3:** Evaluation of RDTs in Experiment 2

		IC kit results				
Exp.2	PCR results	+	–	Sensitivity(%)	Specificity(%)	OAA(%)^a^
TKK-1st	+	93	1	98.9	97.6	98.3
(*n* = 177)	–	2	81			
TKK-2nd	+	93	1	98.9	97.6	98.3
(*n* = 177)	–	2	81			
SD Ag	+	81	13	86.2	98.8	92.1
(*n* = 177)	–	1	82			

^a^*OAA* Overall agreement

*SD Ag* SD Bioline dengue NS1 Ag

Two DENV-PCR-negative samples were detected as false positives by the TKK-1st kit and -2nd kit in Experiment 2 (S-1293 and S-1506). Among 177 samples tested in Experiment 2, 135 samples were IgM-, IgG-negative, but 42 samples were IgM- and/or IgG-positive. When we focused on the 135 IgM-, IgG-negative samples, specificity was 100%, as shown in Table 4. There were 12 samples that were positive for IgM only, 23 samples that were positive for IgG only, and 7 samples that were positive for both IgM and IgG. On the other hand, the sensitivities of the TKK-1st and -2nd kits were not affected by the existence of IgM and/or IgG in the specimens of Experiment 2.

**Table 4:** Evaluation of RDTs in IgM-, IgG-negative samples in Experiment 2

		IC kit results				
Exp.2	PCR results	+	-	Sensitivity (%)	Specificity (%)	OAA (%)*
TKK-1st	+	77	1	98.7	100	99.3
(n=135)	-	0 57				
TKK-2nd	+	77	1	98.7	100	99.3
(n=135)	-	0 57				
SD Ag	+	70	8	89.7	100	93.9
(n=135)	-	0 57	(n=135)			

*OAA: Overall agreement

SD Ag: SD Bioline dengue NS1 Ag

When we combined the data obtained from Experiments 1 and 2, the TKK-1st and -2nd kits demonstrated sensitivities of 96.0% (145/151) and 96.7% (146/151), respectively, while the SD Bioline dengue NS1 Ag kit exhibited a sensitivity of 84.1% (127/151). The specificities of the TKK-1st and -2nd RDT kits were both 98.0% (98/100), while that of the SD Bioline kit was 99.0% (99/100) (Table 5). The raw data can be found in Additional files 4 and 5.

**Table 5:** Evaluation of RDTs in the present study

		IC kit results				
Total	PCR results	+	–	Sensitivity(%)	Specificity(%)	OAA(%)^a^
TKK-1st	+	145	6	96.0	98.0	96.8
(*n* = 251)	–	2	98			
TKK-2nd	+	146	5	96.7	98.0	97.2
(*n* = 251)	–	2	98			
SD Ag	+	127	24	84.1	99.0	90.0
(*n* = 251)	–	1	99			

^a^*OAA* Overall agreement

*SD Ag* SD Bioline dengue NS1 Ag

When we stratified the results by DENV serotype, all RDTs demonstrated sensitivities of 100% for DENV-1 and DENV-3 cases, while the sensitivities of the TKK-1st and -2nd kits and SD Bioline NS1 Ag kit were 98.5, 99.2, and 86.2% (respectively) for DENV-2. In contrast, the sensitivities of TKK-1st and -2nd kits and SD Bioline NS1 Ag kit were 63.6, 63.6 and 45.5%, respectively, in samples for which we failed to determine DENV serotype, a challenge that presumably was due to the lower virus titers in these samples.

The TKK kits detect DENV NS1 proteins but cannot detect anti-DENV antibodies. Therefore, if DENV-infected patients go to the hospital late after the onset of fever, our kits may not detect DENV infection. In contrast, the SD Bioline Duo kit detects IgM and IgG against DENV as well as the DENV NS1 antigen. However, even after considering the detection of both DENV antigen and antibody, the sensitivity of the SD Bioline Duo kit was 90.7% (137/151).

### Comparison with NS1 ELISA

**Fig. 6:**
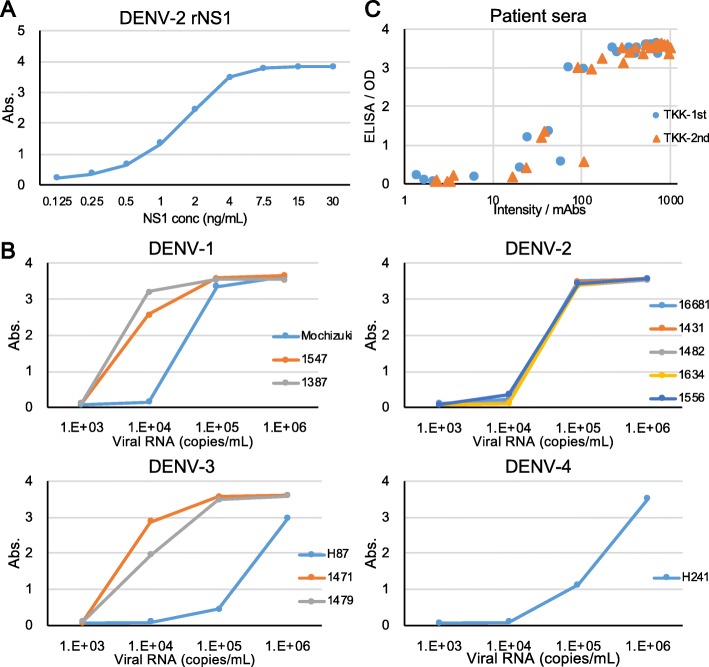
Detection of NS1 protein by ELISA. **a** Serially diluted recombinant NS1 proteins were applied to ELISA. **b** Culture supernatants of Vero cells infected with the indicated virus strains were serially diluted and applied to ELISA. **c** Correlation between ELISA and RDTs. The x-axis indicates color intensities (mAbs: milli absorbance) of TKK-1st kit (blue circles) and TKK-2nd kit (red triangles) and the y-axis indicates optical densities at 450 nm of ELISA from 29 DENV-PCR positive samples

As described above, two DENV-PCR-negative samples yielded false-positive results with the TKK-1st kit and TKK- 2nd kit, while one of these samples also yielded false-positive results with the SD Bioline kit (Table 5). All of these false-positive samples tested positive for anti-DENV IgM and/or IgG antibodies. IgM against DENV are generated at four to seven days after the onset of illness [10, 20, 31]. It is known that NS1 proteins persist longer than the viral genome in blood. Therefore, these false positives most likely were caused by residual NS1 proteins without DENV RNA in patient specimens [20]. To know whether residual NS1 proteins actually existed in these false positive samples, and to further compare the performance of TKK kits with other NS1 detection system, we carried out NS1 antigen ELISA. First, we measured two-fold serially diluted recombinant NS1 protein of DENV-2. When the ISR was above 1, the sample status was determined as positive. We found the detection limit of ELISA was 0.125 ng/mL of recombinant NS1 (Fig. 6a), which was 10 times lower than that of RDTs (2 ng/mL). When we applied the culture supernatant of isolated viruses, the InBios ELISA kit could detect 1 × 10^4^ copies/mL dilution of most of the isolates except for H87 (DENV-3) and H241(DENV-4) (Fig. 6b). These results are similar to those of TKK kits (Fig. 3). Finally, we selected 29 DENV-PCR positive samples with various Ct values including 5 samples which were not detected by RDTs, together with 21 DENV-PCR negative samples including IgM or IgG positive samples. The two false positive samples were also included in these sample set. Among this set of 50 samples (Fig. 6c), sensitivities of TKK-1st and -2nd kits were 82.8% (24/29) and 86.2% (25/29), respectively, while that of the ELISA was 89.7% (26/29). Specificities of TKK-1st and -2nd kits were 90.5% (19/21) while that of the ELISA was 85.7% (18/21). These results suggested that there was no big difference in the sensitivity and specificity between InBios NS1 ELISA and TKK RDTs. In addition, we observed high levels of correlation between optical density of ELISA and color intensity of TKK kits, since Pearson’s Correlation coefficient was 0.83 for TKK-1st and 0.82 for TKK-2nd. Furthermore, the two false positive samples were also detected by the ELISA, indicating that these two samples were in fact NS1 positive (Additional file 5).

### Sequence analysis of NS1-encoding region

To understand sequence variations of DENV in Bangladesh, which might affect the sensitivities of the RDTs, we used RT-PCR to amplify (from stored extracted RNA) the NS1-encoding region; we determined nucleotide sequences for amplicons from 54 samples including 7 DENV-1, 40 DENV-2, and 7 DENV-3 samples. Phylogenetic analysis of the sequencing results revealed that the current circulating viruses were DV1 genotype V (Additional file 6), DV2 genotype cosmopolitan (Additional file 7), and DV3 genotype I (Additional file 8). The reference sequences used in phylogenetic analysis are listed in Additional file 9; sequences of the NS1-encoding regions obtained in the present study are listed in Additional file 10.

**Fig. 7:**
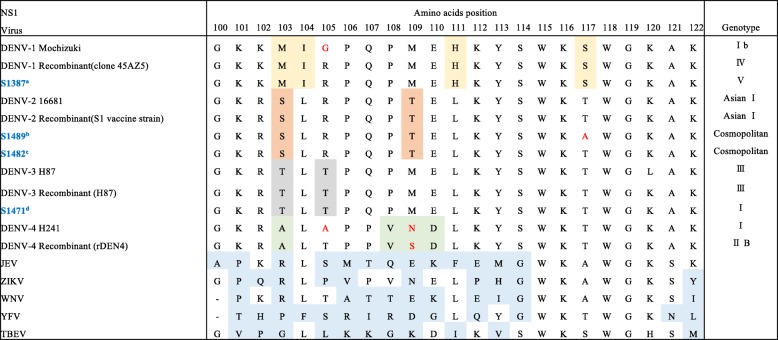
Amino acid variation in positions 100 to 122 of the NS1 protein. Blue color denotes the dengue virus (DENV) obtained in Bangladesh in 2017.^a^All the other DENV-1 sequences determined in the present study had the same sequence as S1387 in positions 100–122.^b^All the other DENV-2 sequences determined in the present study (except for S1482, S1431, and S1285) had the same sequence as S1489 in positions 100–122.^c^S1431 and S1285 had the same sequence as S1482 in positions 100–122.^d^All the other DENV-3 sequences determined in the present study had the same sequence as S1471 in positions 100–122.Yellow, orange, gray, and green cells indicate the DENV-1, DENV-2, DENV-3, and DENV-4-specific amino acids, respectively. Blue cells indicate amino acids that differ from those of DENV. Red colors denote amino acid variation within the serotype. ZIKA: Zika virus, MR766 strain, Q32ZE1 JEV: Japanese encephalitis virus, Nakayama strain, ABQ52691 WNV: West Nile virus, 2002 epidemic strain, AAV54504 YFV: yellow fever virus, French neurotropic strain, AAA99712 TBEV: tick-bone encephalitis virus, 263 strain, AAA86739

There were a few amino acid variations at positions 100–122 of NS1, the region recognized by three out of four of the MAbs that were used in the TKK-1st and -2nd kits (Fig. 7). The TKK-1st kit and -2nd kit failed to recognize 6 and 5 DENV-PCR-positive samples, respectively. We tried to determine NS1 amino acid variations for the NS1 proteins encoded by these viruses, but were unable to amplify the NS1-encoding regions of these samples due to relatively high Ct values.

## Discussion

Although many real-time PCR kits for DENV diagnosis are commercially available at present, these molecular testing kits remain unavailable in many geographic areas where DENV is endemic. Additionally, real-time PCR methods have very slow turnaround times, and are prohibitively expensive in resource-limited environments. However, it is necessary to identify specific pathogens in many infectious diseases, especially those caused by arthropod-borne (mosquito- and tick-transmitted) viruses, given the markedly different risk profiles among these diseases. Therefore, resource-limited regions urgently need RDTs that are inexpensive, easy to use, do not require additional equipment, show immediate results, and can be used at all levels of healthcare systems. The present study evaluated two newly developed RDTs and one commercially available SD Bioline dengue NS1 for their utility in the diagnosis of acute dengue infection in a hospital setting. As reported here, our RDTs demonstrated excellent sensitivity and specificity, with sensitivity exceeding that of the SD Bioline kit in all cases, including laboratory-cultured viruses, clinical viral isolates, and sera from DENV-infected patients.

Our kits incorporated 4 MAb clones. Among these 4 MAbs, one did not react with NS1 protein by western blot, suggesting that this MAb recognizes a conformational epitope. The MAbs in the TKK-1st kit recognized a linear epitope (located at amino acids 100 to 122 of NS1) as well as a conformational epitope of NS1, while both MAbs in TKK-2nd kit recognized linear epitopes (located at amino acids 100 to 122 of NS1) only. We analyzed amino acid sequences at positions 100 to 122 in flaviviral NS1 proteins (Fig. 7). Amino acids 100 to 122 in DENV NS1 are predicted to form a wing domain, are hydrophilic, and shows low levels of identity between DENV and other flaviviruses (Fig. 7). Generally, DF-endemic areas overlap those of Zika fever, and include regions inhabited by *Aedes aegypti* and *Aedes albopictus* mosquitos*,* insects known to transmit these viruses to humans [40]. These closely related co-circulating viruses cause similar clinical symptoms. Therefore, RDTs with high levels of sensitivity and no cross-reactivity would provide useful information for clinical diagnosis in such areas.

Recently, Bosch et al. [41] developed a RDT for DENV serotyping. Those authors used MAbs against epitopes in the latter half of the amino acid 100–122 region as pan-DENV antibodies [41]. This position has been reported to be conserved among DENV serotypes [42]. Therefore, it is reasonable to expect that our kits would show high levels of sensitivity against various genotypes of DENV-1, DENV-2, and DENV-3, and lack cross-reactivity against other flaviviruses. In fact, our kits demonstrated high levels of sensitivity to various DENV strains and serotypes without cross-reactivity against JEV and ZIKV. On the other hand, the SD Bioline dengue NS1 kit demonstrated cross-reactivity to both JEV and ZIKV. We did not evaluate our RDTs with DENV-4-infected serum, because DENV-4 infection was not found in the patient population examined in the present study. However, the DENV-NS1 sequences in amino acid positions 100 to 122, especially in the latter half of this region, showed high levels of identity among all serotype of DENVs including DENV-4 (Fig. 7). We therefore expect that our RDTs also will show high levels of sensitivity against DENV-4 patient sera. Notably, if human antibodies directed against amino acid positions 100–122 of NS1 are generated in infected patients, the TKK-2nd kit would be more affected than the TKK-1st kit.

The median Ct value of the 269 total cases examined in the present study was similar to that of the cases examined in Experiment 1. Therefore, it is reasonable to expect that the kit sensitivity shown in Experiment 1 will be reproducible in clinics similar to Apollo Hospitals Dhaka. In contrast, the median Ct value of the cases examined in Experiment 2 was lower than that of the cases examined in Experiment 1 (Additional file 3). This observation presumably was the reason why kit sensitivity was higher in Experiment 2 than in Experiment 1.

**Fig. 8:**
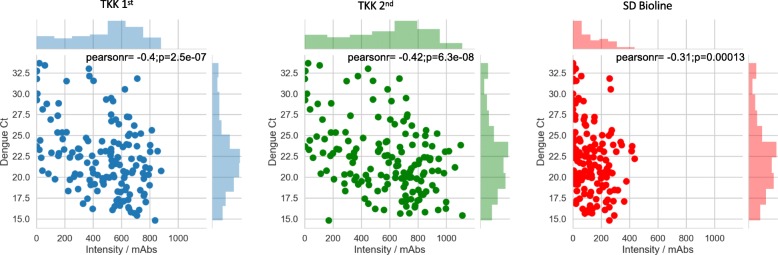
Correlation between color intensity of RDTs and Ct values of DENV detection PCR. Upper and right column bar graphs show distributions of color intensities and Ct values. A correlation coefficient and *p* value for each dot plot is shown in the upper area of each dot-plot graph. The left panel (blue color) is for the TKK-1st kit. The center panel (green color) is for the TKK-2nd kit. The right panel (red color) is for the SD Bioline kit

We further analyzed the correlation between color intensity of the RDTs and the Ct values obtained in the present study (Fig. 8). Simonnet et al. reported that the results of the SD Bioline kit were difficult to judge because of the occurrence of faint bands [24]. The color intensities provided by the TKK-1st kit and -2nd kit were apparently higher than those of the SD Bioline kit, meaning that the TKK-1st kit and -2nd kit should be easier to read and are expected to detect NS1 proteins at later time points post-infection than possible with the SD Bioline kit, given that the TKK kits can detect lower NS1 concentrations in patient sera.

Generally, the sensitivity of RDTs (immunochromatograph format) is affected by the presence of patient-generated antibodies [20, 23, 26, 30, 31]. Although the sensitivities of the TKK-1st kit and TKK-2nd kit were affected by the presence of IgG and/or IgM in patient sera in Experiment 1, sensitivities among IgG- and/or IgM-positive samples were still 80.0% (12/15) in Experiment 1 and 100% (16/16) in Experiment 2. An apparent lack of IgG and/or IgM effects on the results of the TKK-1st kit and TKK-2nd kit in Experiment 2 most likely is explained by the fact that we did not include samples with low viral load in Experiment 2. Nevertheless, the sensitivity of SD Bioline dengue NS1 Ag kit (89.7%) was still affected by anti-DENV IgG and/or IgM in Experiment 2 (68.8%), confirming that the TKK-1st kit and TKK-2nd kit showed higher sensitivities than the SD Bioline dengue NS1 Ag kit.

Unfortunately, the sample collection day relative to the onset of illness was not available for the clinical samples used in the present study. This is an apparent weakness of the present study. We speculate that our RDTs can detect NS1 protein until high concentrations of anti-DENV IgM are generated in patients. An anti-DENV IgM antibody-based assay limited to acute-phase samples would not be sufficient for the diagnosis of DENV infection, since in the majority of DENV infection cases, anti-DENV IgM levels become detectable between day 4 and day 7 after the onset of illness [43–45]. For reliable diagnosis of DENV infection, NS1 antigen-based RDTs would provide useful information in the acute phase, especially in the viremia period, while anti-DENV antibody-based RDTs would be useful in the convalescent phase [10]. In the present study, our RDTs demonstrated that sensitivities were 96.0% with the TKK-1st kit and 96.7% with the TKK-2nd kit. Specificities were 98.0% with both the TKK-1st kit and TKK-2nd kit, and overall agreement with an RT-PCR based assay was 96.8% with the TKK-1st kit and 97.2% with the TKK-2nd kit. These values would be lower if the study was conducted in a tertiary hospital, where severe cases are admitted. Ideally, a combination of our novel NS1 detection kit with RDTs that detect antibodies against DENV without cross-reactivity against other flaviviruses would be employed.

Another weakness of the present study is lack of accurate information regarding the status of infection. One hundred and thirty-two out of 151 DENV PCR positive samples (87.3%) lacked anti-DENV IgG and were theoretically judged as primary infection. However, considering the high levels of DENV seroprevalence in this area [32], it is highly unlikely that all of these 132 cases were in primary infection. As described above, we do not know the sample collection day relative to the onset of illness. However, it is possible that many of these cases came to the hospital at earlier stage of illness before expansion of DENV IgG-secreting plasma cells, since many of the patients have high levels of DENV RNA (Additional file 4). Nevertheless, even among 19 cases with DENV IgG, the sensitivity of TKK-1st kit and TKK-2nd kit were still 84.2% (16/19).

In addition, it has been reported that the sensitivities of many commercially available RDTs against DENV-4 are lower than those against DENV-1, DENV-2, and DENV-3 [15, 25]. The present study did not include any DENV-4 infected patients, but we did show that the sensitivities of the TKK kits were higher than that of the SD Bioline when tested against recombinant DENV-4 NS1 protein (Fig. 2) and against a DENV-4 laboratory strain (Fig. 3). We are currently performing an evaluation of our new kits in hospitals in other countries where DENV-4 is in circulation. It is reasonable to expect that the sensitivity to DENV-4 also would be good, since the amino acid variations in amino acid positions 100–122 of NS1 are only rarely seen in different genotypes of DENV-4 (Additional file 11).

Finally, it should be noted that CHIKV-positive sera did not react with our kit, and that we confirmed that our kits lacked cross-reactivity against other arboviruses, including ZIKV and JEV. It will be necessary to evaluate our RDTs in other DENV endemic areas where ZIKV also circulates.

## Conclusions

Our novel DENV NS1-targeting RDTs demonstrated high levels of sensitivity and lacked cross-reactivity against ZIKV and JEV compared with commercially available RDTs.

## Additional files

Additional file 1: Primers used for amplification and sequencing of the NS1-encoding region. (DOCX 14 kb)

Additional file 2: Dot blot and western blot. (A) Dot blot of recombinant NS1 protein. Serially diluted recombinant DENV-2 NS1 protein fixed on nitrocellulose membrane at the indicated concentration (ng/well) was reacted with 2 μg/well of the 1st antibody (1st Mab: #M1, Membrane-1; #M2, Membrane-2; #A1, AuNPs-1; #A2, AuNPs-2) Peroxidase-conjugated anti-mouse IgG was used to detect antibodies bound to NS1 protein. (B) Western blot of recombinant DENV-1 (left) and DENV-2 (right) NS1 protein. Arrows denote NS1 protein detected by MAbs (#M1, Membrane-1; #M2, Membrane-2; #A1, AuNPs-1; #A2, AuNPs-2). (DOCX 197 kb)

Additional file 3: Comparison of Ct values in each experiment. Center lines in boxes and boxes indicate the median and 25/75 percentiles. The whiskers indicate inner boundary points. Diamonds indicate outliers. PCR: Ct values of DENV in 269 total patient samples measured between July 2017 and February 2018 in Apollo Hospitals Dhaka. Bangladesh. Exp. 1: Ct values of DENV in samples used in Experiment 1. Exp.2: Ct values of DENV in samples used in Experiment 2. (DOCX 43 kb)

Additional file 4: Results of RDT evaluation in all DENV RT-PCR-positive samples. (XLSX 23 kb)

Additional file 5: Results of RDT evaluation in all DENV RT-PCR-negative samples. (XLSX 16 kb)

Additional file 6: Sequence analysis of the DENV-1 NS1-encoding region. A maximum-likelihood phylogenetic tree was constructed in the W-IQ-TREE program using ModelFinder, and an ultrafast bootstrap (UFBoot) of 1000 replicates was calculated. TIM2 + F + G4 was chosen as the best-fit model according to Bayesian information criteria. Data include NS1-encoding sequences obtained in the present study (labeled in red) along with sequences of known genotypes obtained from GenBank. The sequences of recombinant NS1 proteins used in the present study are shown in pink. The sequences of laboratory strain Mochizuki are shown in blue. Viral genotypes are indicated to the right. Virus names are shown as the accession number, country, and reported year of each sequence. Numbers on the right of the branches are UFBoot support values exceeding 75%. * indicate specimens from which clinical isolates in Fig. 5 were obtained. (PDF 56 kb)

Additional file 7: Sequence analysis of the DENV-2 NS1-encoding region. A maximum-likelihood phylogenetic tree was constructed in the W-IQ-TREE program using ModelFinder, and an ultrafast bootstrap (UFBoot) of 1000 replicates was calculated. TF + F + G4 was chosen as the best-fit model according to Bayesian information criteria. Data include NS1-encoding sequences obtained in the present study (labeled in red) along with sequences of known genotypes obtained from GenBank. The sequences of recombinant NS1 protein of laboratory strain 16681 are shown in blue. Viral genotypes are indicated to the right. Virus names are shown as the accession number, country, and reported year of each sequence. Numbers on the right of the branches are UFBoot support values exceeding 75%. * indicate specimens from which clinical isolates in Fig. 5 were obtained. (DOCX 377 kb)

Additional file 8: Sequence analysis of the DENV-3 NS1-encoding region. A maximum-likelihood phylogenetic tree was constructed in the W-IQ-TREE program using ModelFinder, and an ultrafast bootstrap (UFBoot) of 1000 replicates was calculated. TIM2 + F + G4 was chosen as the best-fit model according to Bayesian information criteria. Data include NS1-encoding sequences obtained in the present study (labeled in red) along with sequences of known genotypes obtained from GenBank. The sequences of recombinant NS1 protein of laboratory strain H87 are shown in blue. Viral genotypes are indicated to the right. Virus names are shown as the accession number, country, and reported year of each sequence. Numbers on the right of the branches are UFBoot support values exceeding 75%. * indicate specimens from which clinical isolates in Fig. 5 were obtained. (PDF 57 kb)

Additional file 9: Sequences of NS1 region from GenBank used in phylogenetic analysis. (XLSX 13 kb)

Additional file 10: Sequences of NS1 region obtained in this study. (XLSX 11 kb)

Additional file 11: Amino acid sequences of positions 100 to 122 of DENV-4 NS1 proteins. Virus names are shown as the accession number/country/reported year of each sequence. Green cells denote the DENV-4-specific amino acid positions. Red color indicates amino acid variation that the tested recombinant NS1 protein and laboratory strain lacked. * NC 002640-rDV4 is the original amino acid sequence of the recombinant protein tested in the present study. ** KR011349/Philippines/1956 is the same as the H241 laboratory strain tested in the present study. (DOCX 190 kb)

## Data Availability

All data analyzed during this study are included in this published article and its supplementary information files. The datasets generated during the current study are available in the Genbank repository.

## Abbreviations

AuNPs: Gold nano particles
BSA: Bovine serum albumin
CHIKV: Chikungunya virus
DENV: Dengue virus
DF: Dengue fever
DHF: Dengue hemorrhagic fever
ELISA: Enzyme-linked immuno-sorbent assay
JEV: Japanese encephalitis virus
mAbs: Milli-absorbance units
MAbs: Monoclonal antibodies
NS1: Non-structural protein 1
OAA: Overall agreement
PBS: Phosphate-buffered saline
RDT: Rapid diagnosis test
RT: Room temperature
TBEV: Tick-bone encephalitis virus
TCEP: Tris (2-carboxyethyl)phosphine
TMB: 3,3′,5,5′-tetramethylbenzidine
UF Boot: Ultrafast bootstrap
WNV: West Nile virus
YFV: Yellow fever virus
ZIKV: Zika virus
