# Supplementary material for: Evaluation of novel rapid detection kits for dengue virus NS1 antigen in Dhaka, Bangladesh, in 2017
Source: Virol J. 2019 Aug 15;16:102. doi: 10.1186/s12985-019-1204-y (PMC6694664; doi:10.1186/s12985-019-1204-y)
Supplement: Supplementary file 1 — Primers used for amplification and sequencing of the NS1-encoding region. (DOCX 14 kb) [file 12985_2019_1204_MOESM1_ESM.docx]

**Additional File 1. Primers used for amplification and sequencing of the NS1-encoding region.**

| Serotype | Antisense-primers | Sequence 5'-3' | Sense-primers | Sequence 5'-3' |
| --- | --- | --- | --- | --- |
| DENV-1 | d1a13^a^ | CCRATGGCYGCTGAYAGTCT | d1s6^a^ | GGYTCTATAGGAGGRGTGTTCAC |
|  | d1a14* ^,a^ | CCGGAAGCCATGTTGTTTT | d1s6*^, a, #^ | GGYTCTATAGGAGGRGTGTTCAC |
|  |  |  | d1s7S ^#^ | GACCCAAGGAAAAAAGATG |
|  |  |  | d1s8 ^a, #^ | ACAAACAGCAGGGCCRTGGCA |
| DENV-2 | d2a14^a^ | GCCGTGATTGGTATTGATACAGGA | d2s4^a, #^ | GCGAAGAAACAGGATGTTGTTG |
|  | DV2-3921R* | AGTCACTGCCAATTGATACTTTTCC | DV2-1993F*^,^ | ACAGTCAACCCAATTGTGACAGAAA |
|  |  |  | DV2-seq4^b, #^ | RGGTGACACAGCCTGGGA |
|  |  |  | DV2- seq5^b, #^ | GGCCYCAGCCCACTGAGC |
| DENV-3 | d3a14^a^ | ACTGTGATCATTAARTTGTGGGA | d3s5 ^a,^ | TGAACCTCCTTTTGGGGAA |
|  | d3a15*^, a^ | CCCCARAGCRATTCCATT | d3s5*^, a, #^ | TGAACCTCCTTTTGGGGAA |
|  |  |  | d3s6B ^#^ | CCAAAAGACTGGCGACAGC |
|  |  |  | d3s7 ^a, #^ | CATGGGCTATTGGATAGAAAGC |

^a^ Christenbury, J. G., 2010

^b^ Kurosu, T., 2010

* nested PCR primer pairs

^#^ sequencing primer
