# Supplementary material for: Evaluation of novel rapid detection kits for dengue virus NS1 antigen in Dhaka, Bangladesh, in 2017
Source: Virol J. 2019 Aug 15;16:102. doi: 10.1186/s12985-019-1204-y (PMC6694664; doi:10.1186/s12985-019-1204-y)
Supplement: Supplementary file 2 — Dot blot and western blot. (A) Dot blot of recombinant NS1 protein. Serially diluted recombinant DENV-2 NS1 protein fixed on nitrocellulose membrane at the indicated concentration (ng/well) was reacted with 2 μg/well of the 1st antibody (1st Mab: #M1, Membrane-1; #M2, Membrane-2; #A1, AuNPs-1; #A2, AuNPs-2) Peroxidase-conjugated anti-mouse IgG was used to detect antibodies bound to NS1 protein. (B) Western blot of recombinant DENV-1 (left) and DENV-2 (right) NS1 protein. Arrows denote NS1 protein detected by MAbs (#M1, Membrane-1; #M2, Membrane-2; #A1, AuNPs-1; #A2, AuNPs-2). (DOCX 197 kb) [file 12985_2019_1204_MOESM2_ESM.docx]

**Additional File 2. Dot blot and western blot**

1. Dot blot of recombinant NS1 protein

Serially diluted recombinant DENV-2 NS1 protein fixed on nitrocellulose membrane at the indicated concentration (ng/well) was reacted with 2 µg/well of the 1^st^ antibody (1^st^ Mab: #M1, Membrane-1; #M2, Membrane-2; #A1, AuNPs-1; #A2, AuNPs-2) Peroxidase-conjugated anti-mouse IgG was used to detect antibodies bound to NS1 protein.

1. Western blot of recombinant DENV-1 (left) and DENV-2 (right) NS1 protein. Arrows denote NS1 protein detected by MAbs (#M1, Membrane-1; #M2, Membrane-2; #A1, AuNPs-1; #A2, AuNPs-2).
