# Supplementary material for: Evaluation of novel rapid detection kits for dengue virus NS1 antigen in Dhaka, Bangladesh, in 2017
Source: Virol J. 2019 Aug 15;16:102. doi: 10.1186/s12985-019-1204-y (PMC6694664; doi:10.1186/s12985-019-1204-y)
Supplement: Supplementary file 3 — Comparison of Ct values in each experiment. Center lines in boxes and boxes indicate the median and 25/75 percentiles. The whiskers indicate inner boundary points. Diamonds indicate outliers. PCR: Ct values of DENV in 269 total patient samples measured between July 2017 and February 2018 in Apollo Hospitals Dhaka. Bangladesh. Exp. 1: Ct values of DENV in samples used in Experiment 1. Exp.2: Ct values of DENV in samples used in Experiment 2. (DOCX 43 kb) [file 12985_2019_1204_MOESM3_ESM.docx]

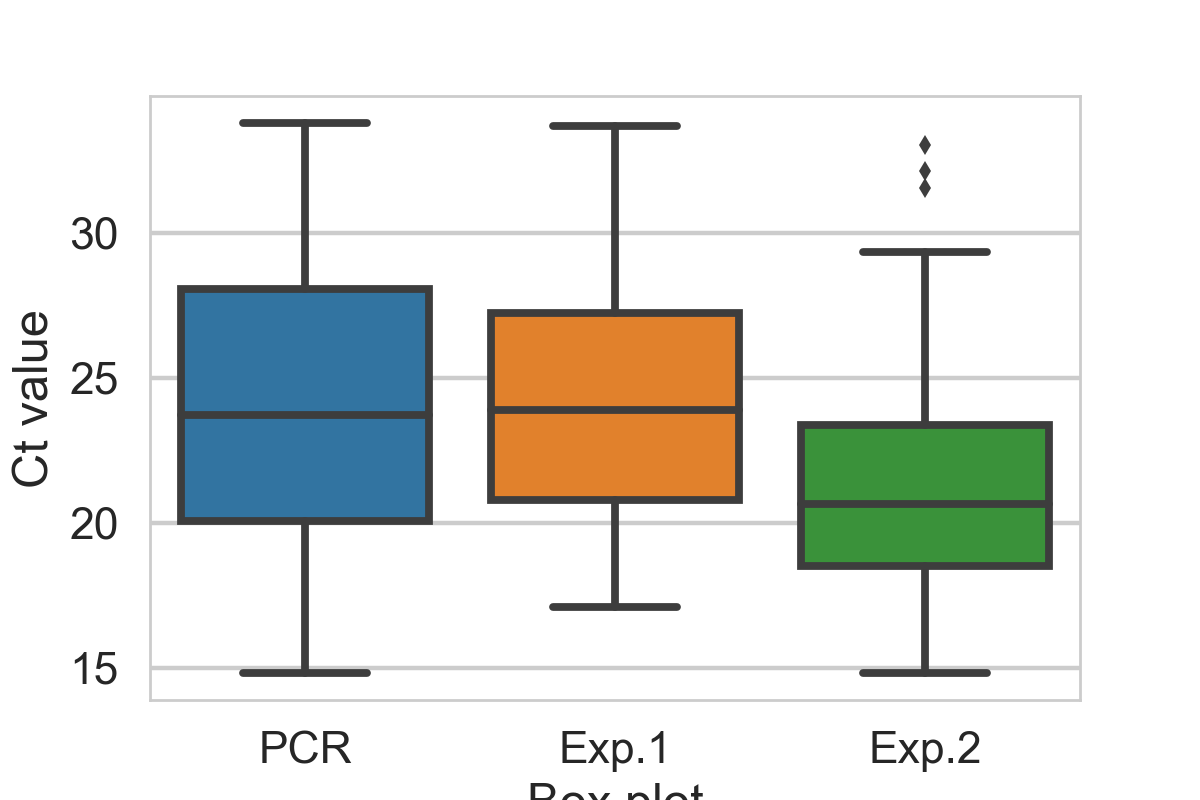


**Additional File 3. Comparison of Ct values in each experiment.**

Center lines in boxes and boxes indicate the median and 25/75 percentiles. The whiskers indicate inner boundary points. Diamonds indicate outliers. PCR: Ct values of DENV in 269 total patient samples measured between July 2017 and February 2018 in Apollo Hospitals Dhaka. Bangladesh. Exp. 1: Ct values of DENV in samples used in Experiment 1. Exp.2: Ct values of DENV in samples used in Experiment 2.
