# Supplementary material for: Evaluation of novel rapid detection kits for dengue virus NS1 antigen in Dhaka, Bangladesh, in 2017
Source: Virol J. 2019 Aug 15;16:102. doi: 10.1186/s12985-019-1204-y (PMC6694664; doi:10.1186/s12985-019-1204-y)
Supplement: Supplementary file 8 — Sequence analysis of the DENV-3 NS1-encoding region. A maximum-likelihood phylogenetic tree was constructed in the W-IQ-TREE program using ModelFinder, and an ultrafast bootstrap (UFBoot) of 1000 replicates was calculated. TIM2 + F + G4 was chosen as the best-fit model according to Bayesian information criteria. Data include NS1-encoding sequences obtained in the present study (labeled in red) along with sequences of known genotypes obtained from GenBank. The sequences of recombinant NS1 protein of laboratory strain H87 are shown in blue. Viral genotypes are indicated to the right. Virus names are shown as the accession number, country, and reported year of each sequence. Numbers on the right of the branches are UFBoot support values exceeding 75%. * indicate specimens from which clinical isolates in Fig. 5 were obtained. (PDF 57 kb) [file 12985_2019_1204_MOESM8_ESM.pdf]

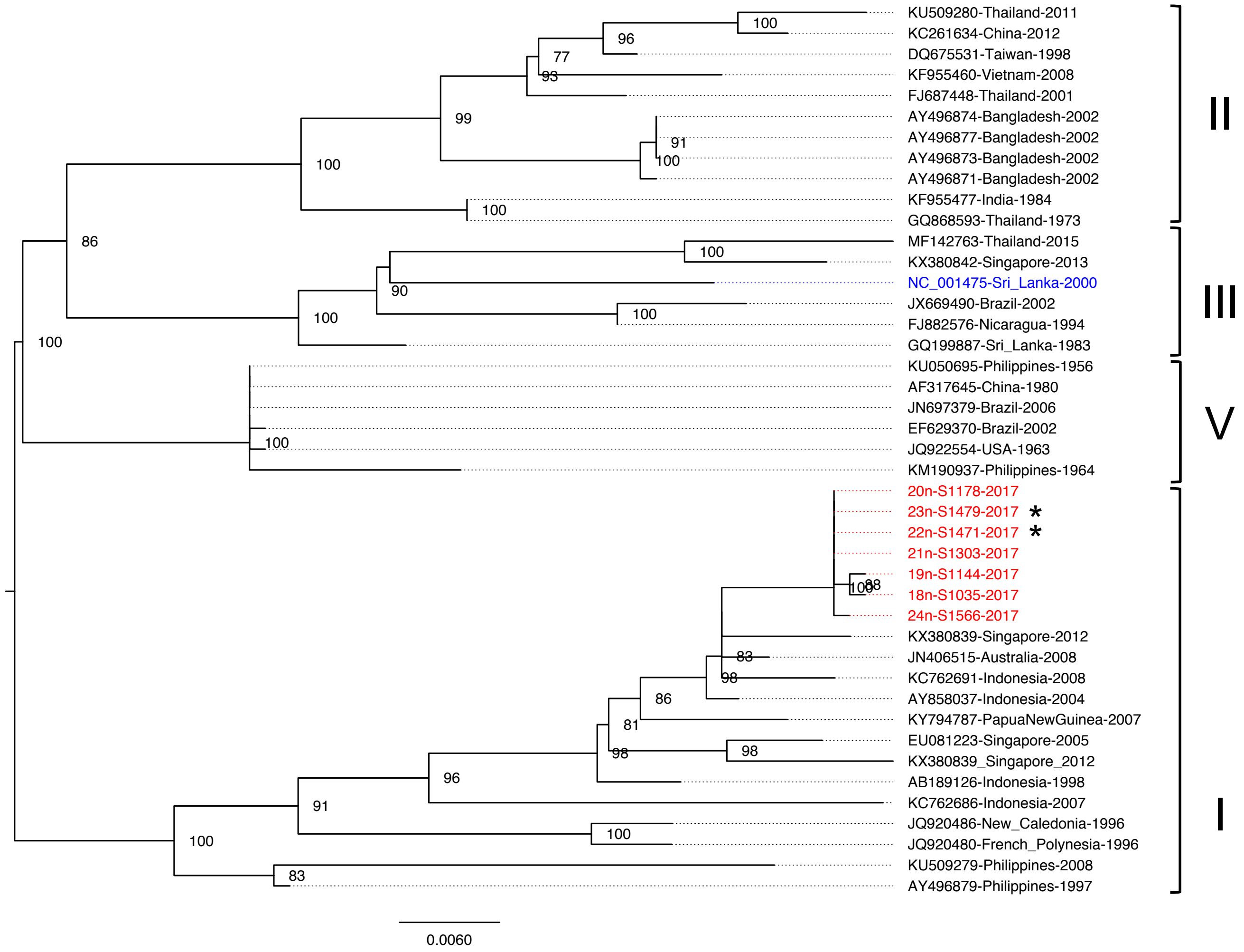

Additional File 8. Sequence analysis of the DENV-3 NS1-encoding region.

A maximum-likelihood phylogenetic tree was constructed in the W-IQ-TREE program using ModelFinder, and an ultrafast bootstrap (UFBoot) of 1000 replicates was calculated. TIM2+F+G4 was chosen as the best-fit model according to Bayesian information criteria. Data include NS1-encoding sequences obtained in the present study (labeled in red) along with sequences of known genotypes obtained from GenBank. The sequences of recombinant NS1 protein of laboratory strain H87 are shown in blue. Viral genotypes are indicated to the right. Virus names are shown as the accession number, country, and reported year of each sequence. Numbers on the right of the branches are UFBoot support values exceeding 75%. * indicate specimens from which clinical isolates in Fig. 5 were obtained.
