# Supplementary material for: Evaluation of novel rapid detection kits for dengue virus NS1 antigen in Dhaka, Bangladesh, in 2017
Source: Virol J. 2019 Aug 15;16:102. doi: 10.1186/s12985-019-1204-y (PMC6694664; doi:10.1186/s12985-019-1204-y)
Supplement: Supplementary file 11 — Amino acid sequences of positions 100 to 122 of DENV-4 NS1 proteins. Virus names are shown as the accession number/country/reported year of each sequence. Green cells denote the DENV-4-specific amino acid positions. Red color indicates amino acid variation that the tested recombinant NS1 protein and laboratory strain lacked. * NC 002640-rDV4 is the original amino acid sequence of the recombinant protein tested in the present study. ** KR011349/Philippines/1956 is the same as the H241 laboratory strain tested in the present study. (DOCX 190 kb) [file 12985_2019_1204_MOESM11_ESM.docx]

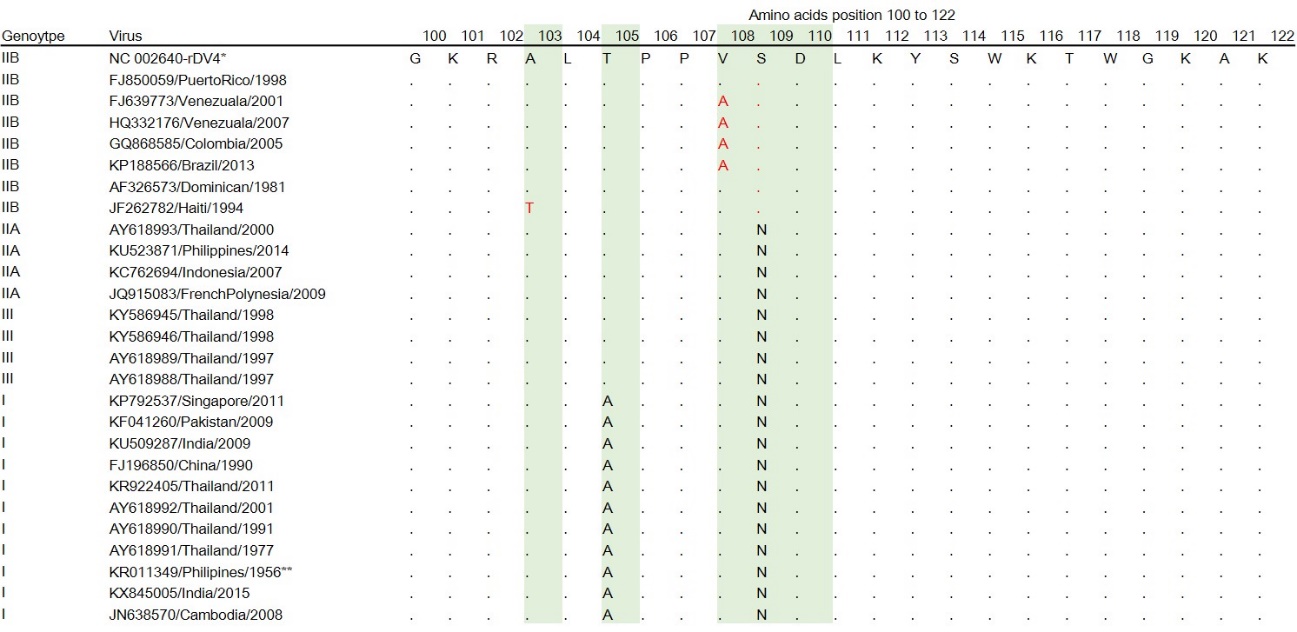


**Additional File 11. Amino acid sequences of positions 100 to 122 of DENV-4 NS1 proteins.**

Virus names are shown as the accession number/country/reported year of each sequence. Green cells denote the DENV-4-specific amino acid positions. Red color indicates amino acid variation that the tested recombinant NS1 protein and laboratory strain lacked.

* NC 002640-rDV4 is the original amino acid sequence of the recombinant protein tested in the present study.

** KR011349/Philippines/1956 is the same as the H241 laboratory strain tested in the present study.
